# Supplementary material for: FGFR2 Mutation p.Cys342Arg Enhances Mitochondrial Metabolism-Mediated Osteogenesis via FGF/FGFR-AMPK-Erk1/2 Axis in Crouzon Syndrome
Source: Cells. 2022 Oct 5;11(19):3129. doi: 10.3390/cells11193129 (PMC9563077; doi:10.3390/cells11193129)
Supplement: Supplementary file 1 [file cells-11-03129-s001.zip › cells-1918229-supplementary.pdf]

FGFR2 Mutation p.Cys342Arg Enhances Mitochondrial Metabolism-Mediated Osteogenesis via FGF/FGFR-AMPK-Erk Axis in Crouzon Syndrome

Mutation Screening

The genetic mutations of two sporadic patients were detected by whole-exome sequencing (WES) conducted by Biomarker Technologies, China, which refers to a genome analysis method that uses sequence capture technology to concentrate DNA in all exon regions of the genome for high-throughput sequencing analysis. Quality control, alignment analysis, and the calling analysis of WES data was shown in Supplementary Table S1 and Table S2. The possible deleterious mutation must be variants with a minor allele frequency <5% as reported in available databases (1000 genomes and esp6500 from NHLBI and EXAc) and alter the original amino acid sequence such as missense mutation, deletion or insertion. To verify the accuracy of whole-exon sequencing results, we utilized Sanger sequencing. Exon 8 and the flanking intronic regions of the FGFR2 gene were amplified in both patients and healthy controls with polymerase chain reaction (PCR). The primer sequences were as follows: forward, 5′- ATGCGTTTGATTGCGTGCAT-3′, and reverse, 5′-TATGCAAGGATAAAAGGGGCCA-3′ (product size 493bp, evaluated by agarose gel electrophoresis shown as Supplementary Figure S1). PCR products were sequenced in both forward and reverse directions by Tsingke Biotechnology Co. Ltd. China.

Pathogenic analysis

We used the T-COFFEE Multiple Sequence Alignment Server (https://tcoffee.crg.eu/) to align the orthologous sequences of FGFR2 downloaded from UniProt (http://www.uniprot.org): Homo sapiens, Mus musculus, Rattus norvegicus, Cavia porcellus, Pan troglodytes, Equus caballus, Papio anubis, and Oryctolagus cuniculus. The PSIPRED online server (http://bioinf.cs.ucl.ac.uk/psipred) and SWISS-MODEL online server（https://swissmodel.expasy.org）were applied to construct the 3D structures of the wild-type and Cys342Arg mutant FGFR2. Predicted structures referred to the structure displayed in PyMol software. The possible impact on the structure and function of the corresponding protein was predicted by sorting intolerant from tolerant (SIFT, PolyPhen-2, Mutation Assessor), as shown in Supplementary Table S3.

Supplementary Table S1. Quality control and alignment analysis of WES data.

Sample	Total effective yield (Gb)	Average sequencing depth	Q20%	Q30%	Mapping rate on genome (%)	Coverage of exome (%)	Fraction of target covered≥4x(%)	Fraction of target covered≥20x (%)
**Patient A**	15.77	159.38	97.22	92.97	99.65	99.9	99.8	98.3
**Patient B**	18.64	218.8	97.97	94.19	99.92	99.7	99.5	98.1

Supplementary Table S2. Calling analysis of WES data.

Sample	SNPs	Exonic	Intronic	Missense	Indels	Frameshift deletion	Frameshift insertion	Stoploss	Stopgain
**Patient A**	243976	24322	90314	11470	36353	148	110	1	8
**Patient B**	199592	24251	97693	11532	29194	143	110	2	8

Supplementary Table S3. The results of three kinds of variation prediction software show that FGFR2 c.1024T>C p.Cys342Arg is likely to cause alterations in protein structure or function.

Prediction software	Prediction score	Reference score
**SIFT**	**0.00**	**≤0.05**
**PolyPhen-2**	**0.997**	**≥0.909**
**Mutation Assessor**	**4.15**	**≥1.9**

Supplementary Table S4 Primer pairs used in qRT-PCR analysis.

Gene	Sense primer	Antisense primer	Product size (bp)
Rps18	AGTTCCAGCACATTTTGCGAG	TCATCCTCCGTGAGTTCTCCA	166
Fgfr2	AATCTCCCAACCAGAAGCGTA	CTCCCCAATAAGCACTGTCCT	142
ColIα2	TCGTGCCTAGCAACATGCC	TTTGTCAGAATACTGAGCAGCAA	222
Runx2	GACTGTGGTTACCGTCATGGC	ACTTGGTTTTTCATAACAGCGGA	84
Alp	CCAACTCTTTTGTGCCAGAGA	GGCTACATTGGTGTTGAGCTTTT	110
Ocn	TCTGACAAAGCCTTCATGTCC	AAATAGTGATACCGTAGATGCG	199
Opn	TGCACCCAGATCCTATAGCC	TGTGGTCATGGCTTTCATTG	150
Mfn2	AGAACTGGACCCGGTTACCA	CACTTCGCTGATACCCCTGA	82
Opa1	CAGCTGGCAGAAGATCTCAAG	CATGAGCAGGATTTTGACACC	107
Drp1	GCAAGAGAACTACCTTCCGCTG	GTTGTCGGTTCCTGACCACCAT	86

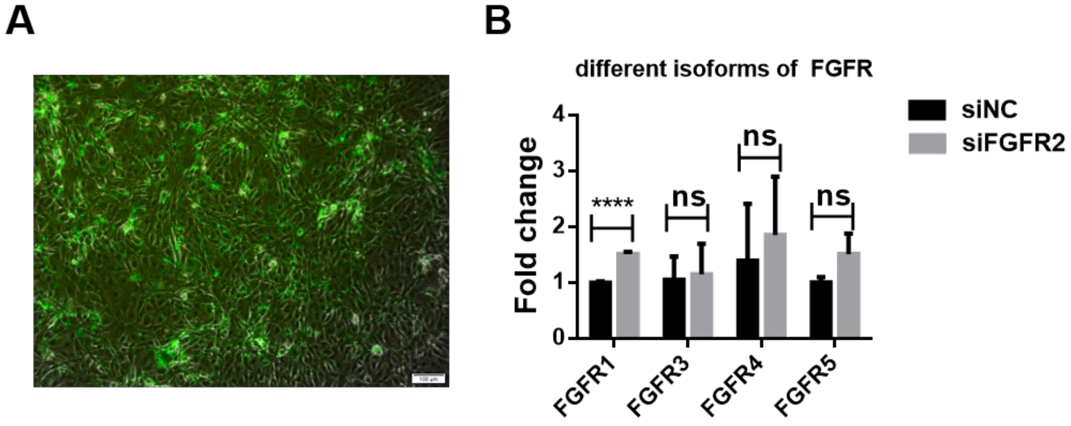

Supplementary Figure S1. (**A.**) Transfection efficiency of siRNA labeled with FAM was detected by fluorescence microscopy. (**B.**) The knockdown efficiency of siRNA was examined via qRT-PCR. The expression of other types *Fgfr*, *Fgfr1*, *Fgfr3*, *Fgfr4* and *Fgfr5* was not decreased at the transcriptional level after the knockdown of *Fgfr2* by specific siRNA.

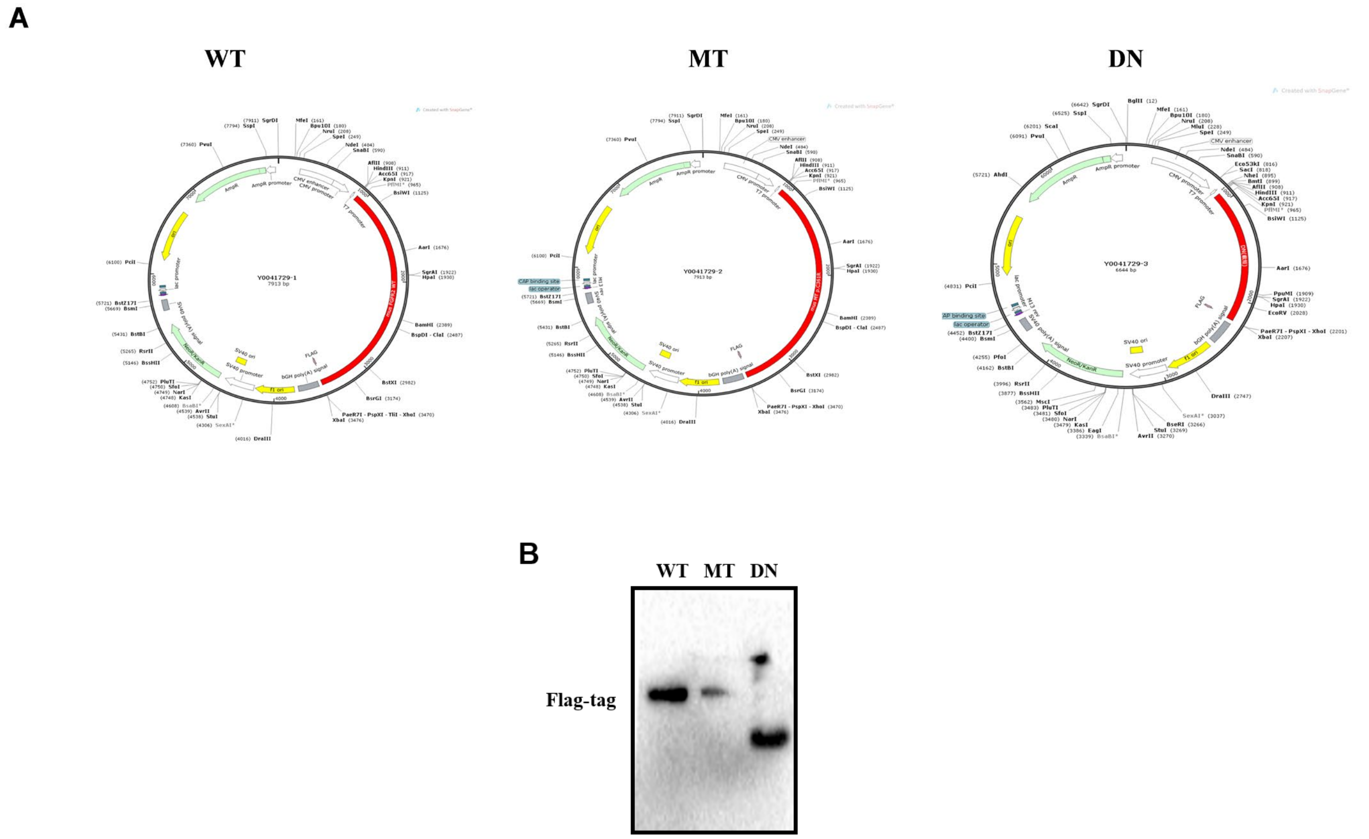

Supplementary Figure S2. (**A.**) The maps of the vector with target genes, the wild-type *Fgfr2*, p.Cys342Arg mutant *Fgfr2* and domain negative (DN) *Fgfr2*. The sequence of DN was reserved at the mutation site p.Cys342Arg but did not have the sequence of the intracellular tyrosine kinase domain that differed from the mutant plasmid. The Flag-tag was on the side of C-terminal. (**B.**) The label Flag-tag was examined by Western blot to examine the transfection efficiency. The molecular weight of domain negative FGFR2 (DN) was smaller because of the truncated intracellular domain.

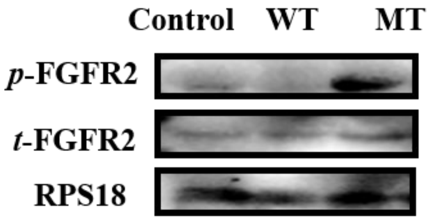

Supplementary Figure S3. Empty vector transfected into MC3T3-E1 cells as control: Western blot showed that there was no significant difference in the level of *t*-FGFR2 and *p*-FGFR2 between the control and WT group, but the level of *p*-FGFR2 was increased in the MT group.

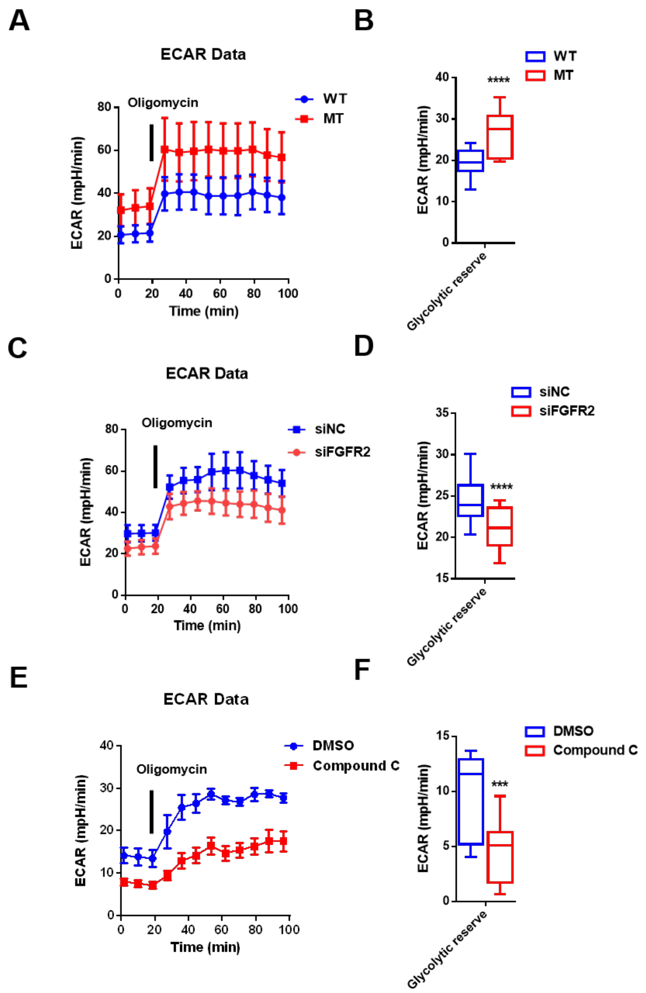

Supplementary Figure S4. Glycolytic function elevated by the activation of AMPK. First platform of ECAR shows acid production by basic glycolysis added with glucose. After treated being with oligomycin, because of the inhibition of oxidative phosphorylation, osteoblastic precursor cells MC3T3-E1 were forced to complete glycolysis for the energy supply, reflecting glycolytic capacity. (**A.**) There was an improvement in the glycolytic reserve of the MT group. (**B.**) ECAR of glycolytic reserve in MT group was 26.58 ± 5.53 mpH/min while that in the WT group was 19.19 ± 3.68 mpH/min. (**C.**) Glycolysis function was attenuated in the siFGFR2 group. (**D.**) ECAR of glycolytic reserve was 21.13 ± 2.38 mpH/min in siFGFR2 group vs. 24.59 ± 2.61 mpH/min in siNC group. (**E.**) After being treated with Compound C, glycolysis function was inhibited. (**F.**) The ECAR of the glycolytic reserve was decreased to some extent in the Compound C group, which was 4.63 ± 2.55 mpH/min, and less than half of that in DMSO group, 10.07 ± 3.46 mpH/min.

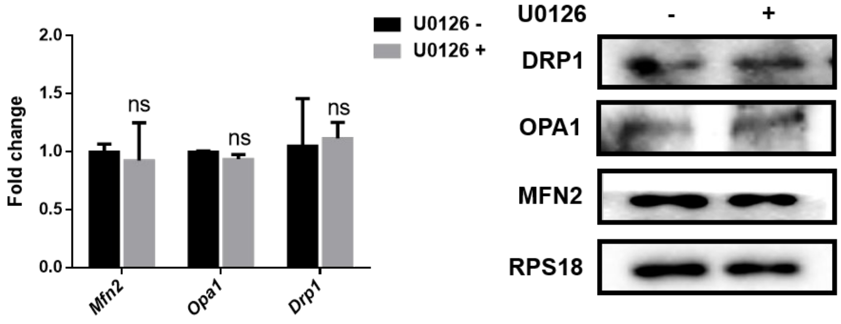

Supplementary Figure S5. The expression of mitochondrial dynamic-related genes *Mfn2*, *Opa1*, *Drp1* had no significant changes after the cells were treated with U0126, indirectly indicating that the Erk signal was downstream from the AMPK signal.
